# Displaced Femoral Neck Fractures Treated with Percutaneous Compression Plates in Elderly Individuals: An Effect Analysis Based on Imaging

**DOI:** 10.2174/0115734056349481241218062504

**Published:** 2025-01-02

**Authors:** Huli Liu, Kai Zhao, Ying Yang, Liansheng Dai, Sanjun Gu, Haifeng Li, Yu Liu

**Affiliations:** 1 Department of Radiology, Wuxi Ninth People’s Hospital Affiliated to Soochow University, Wuxi 214062, Jiangsu, China; 2 Department of Orthopaedics, Wuxi Ninth People’s Hospital Affiliated to Soochow University, Wuxi 214062, Jiangsu, China; 3 Department of Orthopaedics, Jiangnan University Affiliated Hospital, Wuxi 214000, Jiangsu, China

**Keywords:** Femoral neck fracture, Fixation, internal, Percutaneous compression plate, Elderly, Efficacy

## Abstract

**Background:**

The effects of percutaneous compression plate (PCP) internal fixation for femoral neck fractures (FNFs) in elderly individuals have rarely been reported. Therefore, this study aimed to investigate the efficacy of PCCP internal fixation for displaced FNFs in elderly individuals based on imaging.

**Methods:**

The clinical data of 32 elderly patients with FNFs treated with PCCP from January 2015 to December 2022 were retrospectively analyzed. The average age of the participants was 68.7 ± 4.8 years (range, 65–80 years). Nineteen patients had Garden type III, and 13 patients had Garden type IV. Six patients had Pauwels type I, 15 patients had type II, and 11 patients had type III. Twelve patients had Singh index level IV, 14 patients had level V, and 6 patients had level VI. The time from injury to operation ranged from 3–14 days, with an average of 5.8 days. A radiological assessment was conducted. The relationships between efficacy and age, Pauwels classification, the Singh index, and the Garden alignment index were analyzed.

**Results:**

At postoperative week 1, fracture reduction was acceptable in 31 patients. The time to start walking was 5.7 ± 3.7 days. The follow-up time ranged from 2.1 to 4 years, with an average of 2.7 years. There were 2 cases of delayed healing and no cases of nonunion or internal fixation failure. The healing time ranged from 4–8 months, with an average of 4.9 months. Fifteen patients (46.9%) showed healing with shortening of the femoral neck, and 3 patients (9.4%) had avascular necrosis (AVN). Correlation analysis revealed that healing with shortening of the femoral neck was positively correlated with age and the Singh index and that AVN was positively correlated with the Pauwels classification (*p* < 0.05).

**Conclusion:**

The efficacy of PCCPs for internal fixation of displaced FNFs in elderly individuals without severe osteoporosis is satisfactory, especially for patients who can ambulate early postoperatively. The main complications are healing with shortening of the femoral neck and AVN, which are prone to occur in patients with severe osteoporosis and Pauwels type III FNFs, respectively.

## 1 INTRODUCTION

As the proportion of aging adults increases worldwide, the incidence of hip fractures has correspondingly increased. Hip fractures among elderly individuals are often caused by a low-energy injury, which results in impaired independence and quality of life and is associated with a 30% mortality rate at one year; thus, hip fracture is also sometimes referred to as the last fracture in life [1]. Approximately 50% of all hip fractures are femoral neck fractures (FNFs). Early surgery is recommended for the treatment of FNFs in elderly patients as long as there is no obvious contraindication for surgery. There are two operation options for FNFs in elderly individuals, *i.e*., hip arthroplasty and internal fixation, each of which has advantages and disadvantages. The incidence of nonunion and avascular necrosis (AVN) of the femoral head has been reported to be 15%-35% after conventional internal fixation *via* cannulated screws (CSs) and dynamic hip screws (DHSs) and 10%-20% after prosthetic loosening, rupture, dearticulation, periprosthetic fractures, postoperative hip infection, and hip pain after arthroplasty [2-8]. Therefore, internal fixation is the first choice for relatively young patients (< 65 years old) with FNFs, whereas arthroplasty is the first choice for older patients (>70 years old) with displaced FNFs. However, the optimal surgery for those who are 65-70 years of age remains unclear. In recent years, the complication rates associated with new devices for internal fixation of FNFs, such as the dynamic compression locking system, the percutaneous compression plate (PCCP), the Targon-FN, PH intramedullary nails, and the femoral neck system (FNS), have been significantly lower than those associated with traditional CSs and DHSs, among which PCCPs have the lowest nonunion rate (less than 2%). In view of the satisfactory results of PCCPs for internal fixation of FNFs in young and middle-aged patients [9-13], we also used this approach for the treatment of FNFs in some elderly individuals. However, the effect of PCCP on internal fixation of FNFs in elderly patients has not been reported in the literature. Therefore, the purpose of this study was to investigate the efficacy of PCCP insertion for the internal fixation of displaced FNFs in elderly individuals on the basis of imaging.

## 2 MATERIALS AND METHODS

### 2.1 Inclusion and Exclusion Criteria

The inclusion criteria were as follows: (I) patients aged ≥ 65 years, (II) patients with a fresh FNF, (III) patients with a displaced FNF (Garden type III and type IV), (IV) patients who underwent fixation with a PCCP and (V) patients who were followed up for > 2 years postoperatively. The exclusion criteria were as follows: (I) patients with pathological fractures; (II) patients with neck base FNFs; (III) patients with a Singh index level of I-III; (IV) patients who were unable to walk independently before the operation; (V) patients with a poor quality of fracture reduction (Garden alignment index grade III); (VI) patients who underwent treatment with a vascularized bone flap spontaneously; (VII) patients with symptomatic hip osteoarthritis, rheumatoid arthritis, malignant tumors, long-term alcoholism or hormone use; and (VIII) patients with incomplete follow-up data.

### 2.2 Sample Size and Patients

According to the literature, the incidence of complications associated with conventional internal fixation for the treatment of femoral neck fractures is 30%, whereas the incidence of complications associated with PCCP internal fixation is 10%. Taking α= 0.05% and power = 80%, a sample size of 31 cases was determined to be required after calculation.

This retrospective study was reviewed and approved by the Institutional Review Board of our hospitals (No. LW-2024027), and all patients provided written informed consent. All methods were performed in accordance with the ethical standards of the committee responsible for human experimentation (institutional and national) and with the Helsinki Declaration of 1975, as revised in 2013. From January 2015 to December 2022, 38 consecutive elderly patients with displaced FNFs were included at our level 1 trauma centers; 6 patients were excluded, and 32 patients were included. Details of the treatment and follow-up of the patients were prospectively recorded and retrospectively analyzed. There were 15 men and 17 women, with an average age of 68.7 ± 4.8 years (range, 65–80 years). All patients had traumatic fractures, including 14 with fractures that were sustained as the result of a fall, 5 with fractures that were sustained as the result of a crush injury in a traffic accident, and 3 with fractures that were sustained as the result of tripping. With respect to the Garden classification, 19 patients had type III fractures, and 13 patients had type IV fractures. Regarding the Pauwels classification, 6 patients had type I fractures, 15 patients had type II fractures, and 11 patients had type III fractures. Six patients had vertebral fractures or distal radius fractures. Twenty-six patients (81.3%) had medical diseases, including 13 patients with hypertension or coronary heart disease, 8 patients with chronic bronchitis or emphysema, 10 patients with cerebral infarction, 7 patients with diabetes, and 6 patients with severe anemia. There were 15 patients with one disease and 11 patients with two or more diseases. The average time from injury to operation was 5.8 days (range, 3–12 days). The Singh index level of the femoral neck was IV in 12 patients, V in 14 patients, and VI in 6 patients. The bone mineral density analyzed by dual-energy X-ray (DEXA, spontaneously, USA)] was -1.91 ± 0.21 SD (range, -1.0 — -2.6 SD). The Harris hip score (HHS) before the operation was 54. 4 4 ± 6.27.

### 2.3 Surgical Procedure

On admission, skin traction was performed, or a T-shoe was employed for all patients. The routine examinations included whole blood cell counts, urine and biochemical monitoring, an ECG scan, and a chest X-ray examination. Patients with hypertension were treated with antihypertensive drugs to control their blood pressure at approximately 150/90 mmHg; patients with coronary heart disease or/and cerebral infarction were treated with anti-ischemic, antiplatelet or anticoagulation therapy; patients with chronic bronchitis or emphysema were treated with bronchodilators; patients with diabetes mellitus used medication to control their blood glucose below 10 mmol/L; and patients with severe anemia received a blood transfusion to correct their Hb to approximately 80 g/L. After general or spinal anesthesia (17 and 15 patients, respectively), the patients were placed in the supine position on a traction table. Standard anteroposterior (AP) and lateral views were obtained *via* C-arm fluoroscopy to confirm fracture reduction and internal fixator placement. For displaced fractures, satisfactory reduction was usually achieved *via* longitudinal traction and pronation. The PCCP plate was introduced into the lateral aspect of the femur after subperiosteal dissection through a 2.5-cm incision inferior to the greater trochanter. Thereafter, a 3-cm distal incision of the plate was made, and the plate was fixed to the femoral shaft with a bone hook. The distal neck screw was first placed near the calcar femur; then, screws were placed in the proximal, middle, and distal areas of the femoral shaft; and finally, the proximal neck screw was placed.

### 2.4 Postoperative Management

Patients were given antibiotics intravenously for 3 days and anticoagulant therapy for 6 weeks postoperatively. The patients were instructed to ambulate with crutches or walkers within 1 week after the operation until fracture healing was observed *via* radiography. The partial weight-bearing time for patients with Pauwels type III fractures was limited (< 2 h/day) in the early recovery stage.

All patients received an initial monthly follow-up to assess fracture healing, then every 3 months after fracture healing until 1 year after the operation, and every 6 months thereafter. An X-ray examination was routinely performed at each follow-up. If the X-ray was difficult to evaluate regarding fracture healing and the patient complained of coxalgia or if the X-ray films showed suspicious abnormal changes, CT and/or MRI examinations were performed. Eighteen patients were evaluated by CT, and 5 patients were evaluated by MRI in this study.

### 2.5 Evaluation

The operation time, intraoperative blood loss, postoperative hospital stay, time to start walking, fracture healing, internal fixation failure, healing with shortening of the femoral neck, AVN, and functional recovery of the hip joint were recorded. The formula for calculating intraoperative blood loss was as follows: (blood gauze weight - dry gauze weight + suction bottle drainage volume - irrigating fluid weight) × 1.05. Fracture reduction was evaluated *via* the Garden alignment index described by Haidukewych *et al*. [14]. Fracture healing was defined as a fuzzy fracture line and continuous callus passing through the fracture line on the X-ray film and/or CT scans. Nonunion was defined as the persistence of a fracture line for more than 8 months, and delayed healing was defined as occurring more than 6 months before fracture union after the operation. Obvious displacement of the fracture end or varus deformity of the hip (displacement ≥ 2 mm or angulation ≥ 10°) was defined as fixation failure. Healing with shortening of the femoral neck was defined as ≥ 5 mm shortening of the femoral neck central axis compared with the contralateral axis. AVN was diagnosed on the basis of the 2019 Chinese adult expert consensus guidelines [15].

### 2.6 Statistical Analysis

All the statistical analyses were performed *via* SPSS version 23.0 software (SPSS Inc., Chicago, IL, USA). Continuous data are expressed herein as the means ± standard deviations. Binary correlation analysis or the rank sum test was used to analyze the relationships between efficacy (delayed and healing with shortening of the femoral neck, AVN, and function) and age, Pauwels classification, the Singh index, and the Garden alignment index. *p* < 0.05 was considered to indicate a statistically significant difference.

## 3 RESULTS

### 3.1 Perioperative Results

The surgical duration was 73.4 ± 10.3 min (45–135 min). The volume of intraoperative blood loss was 116.4 ± 17.5 ml (75–385 ml). The mean postoperative hospital stay was 8.3 days (3–15 days). According to the Garden alignment index from the X-ray examination at week 1 postoperatively, there were 25 patients with Grade I cases, 6 patients with Grade II cases, and 1 patient with Grade III cases (acceptable in 31 cases). There were no local or systemic complications during the operation. There were also no cases of deep vein thrombosis, pulmonary embolism, or bedsores. The time to start walking was 5.7 ±3.7 days (1–16 days).

### 3.2 Complications and Follow-up

The mean follow-up time was 2.7 years (range, 2.1–4 years). There were 2 cases of delayed union, which involved displaced subcapital or transcervical types of FNFs, and no nonunion or internal fixation failure occurred. The healing time ranged from 4–8 months, with an average of 4.9 months. Fifteen patients (46.9%) showed healing with shortening of the femoral neck, with 5–19 mm shortening in 11 patients and ≥ 20 mm shortening in 4 patients. There were 3 cases (9.4%) of AVN, all of which were displaced subcaptial or transcervical types. Two patients underwent total hip replacement, and the other patient underwent conservative treatment. The HHSs were 87.4 ± 7.6, 89.6 ± 12.8, 89.9 ± 13.2, and 91.0 ± 14.1 at 3 months, 1 year, and 2 years postoperatively and at the last follow-up, respectively, and these differences were statistically significant compared with the preoperative HHS (all *p* < 0.05); there was no significant difference among the postoperative timepoints (*p* > 0.05). At the last follow-up, thirty-one patients (96.9%) could walk independently, 1 patient (3.1%) could not walk independently, and 1 patient with AVN was treated conservatively. Correlation analysis revealed that delayed healing and AVN were not correlated with age, Pauwels classification, the Singh index, or the Garden alignment index (*p* > 0.05), but healing with shortening of the femoral neck was positively correlated with age and the Singh index, and the ANV was positively correlated with the Pauwels classification (*p* < 0.05). Rank sum test analysis revealed that functional recovery was not correlated with age, Pauwels classification, the Singh index, or the Garden alignment index (*p* > 0.05) (Table **1**). A typical case is shown in Fig. (**1**).

## 4 DISCUSSION

### 4.1 Characteristics and Special Requirements for Surgery on FNFs in Elderly Individuals

The incidence of FNFs occurring in combination with chronic medical diseases, including cerebrovascular disease, diabetes, pulmonary disease, and anemia, was 81.3%, of which 46.9% of the cases were combined with one disease and 34.4% of the cases were combined with two or more medical diseases [16, 17]. In elderly patients, being bedridden for long periods exacerbates their underlying diseases and weakens their resistance and immunity [16-18]. Therefore, these patients cannot tolerate complex, long surgeries or multiple surgeries within a short time, and only minimally invasive surgical methods that allow for early walking after surgery are appropriate. Another issue is that elderly individuals, especially elderly women, often have different degrees of osteoporosis, which accounted for 78.1% of the patients in this study. Screw loosening or screw withdrawal and cutting easily occur, and therefore, FNFs in patients with severe osteoporosis are considered contraindications for internal fixation [2, 6, 18]. Thus, severe osteoporosis, not advanced age, is a contraindication for the internal fixation of FNFs [3, 6]. Some surgeons have reported a higher healing rate (80% - 94%) and satisfactory functional recovery of the hip if the FNFs in elderly patients meet the indications for internal fixation [17, 19, 20]. Boraiah *et al*. [19] reported that 54 elderly patients with FNFs (average age 78.1 years) were treated with CSs, and their healing rate was 94%. Ju *et al*. [17] reported that in 73 patients who were older than 65 years and had femoral neck fractures that were treated with CSs, the healing rate was 98.63%, the rate of satisfactory functional recovery was 89%, and the incidence of AVN was 9.6%.

### 4.2 Performance and Effect of PCCPs

Fixation with PCCPs is also a minimally invasive procedure because PCCPs are implanted and fixed through two small incisions. The two main screws of the PCCP that are placed in the femoral head and neck are relatively thick (7.3 mm) and are connected to the steel plate located on the lateral side of the proximal femur at an obtuse angle, which can transfer the bending force of the head and neck screw to the subtrochanteric cortex for fixation. Therefore, PCCPs have stronger anti-compression characteristics, anti-bending resistance, and anti-shear and anti-rotation stability. Brandt *et al.* [21] conducted biomechanical experiments *in vitro* and reported that the antiaxial compression force of a PCCP was 3 times greater than that of a CS and that the combined anticompression and antirotation force of a PCCP was 2 times greater than that of a DHS. Another characteristic of PCCPs is the sliding compression of the two main screws in the head and neck, which can stimulate the fracture ends and is conducive to shortening the healing time of the femoral neck. Therefore, patients with fractures fixed with PCCPs can ambulate and bear weight immediately after the operation, and their union rate is high. Since the use of PCCPs for the fixation of FNFs was first reported in 2008 by Brandt *et al.* [22], they have been used in more than 500 patients; for example, Zhu *et al.* [10] reported 74 cases, Yin *et al*. [25] reported 35 cases, Jin *et al.* [23] reported 51 cases, and Wang *et al*. [24] reported 173 cases. Mukherjee [12] reported the nonunion of FNFs treated with PCCPs and vascularized bone grafts. According to reports of PCCP insertion for FNF in the literature, although the majority of patients were young adults, a small number of elderly patients who were older than 65 years of age were also included. Most patients achieved early walking with weight-bearing after surgery, and the overall fracture healing rate was 98% - 100%, the incidence of healing with femoral neck shortening was 25%-35%, the incidence of AVN was 10%-12%, and the rate of excellent and good outcomes was 90%-98% [10, 12, 13, 23, 24]. The overall outcomes were very satisfactory.

In this study, the time to start walking was 5.7 ± 3.7 days, the healing rate was 100%, the rate of healing with femoral neck shortening was 46.9%, and the excellent and good rates were 84.4%. The general efficacy was satisfactory and similar to that in young and middle-aged patients [10, 12, 13, 15]; in particular, all patients were able to ambulate early postoperatively.

The main complications of fixation with PCCPs for the treatment of displaced FNS in elderly patients are healing with femoral neck shortening and AVN. Healing with shortening of the femoral neck is a frequently observed complication of FNFs after internal fixation. The incidence of healing with femoral neck shortening in young and middle-aged individuals is 25%-35% [10, 12, 23-25], whereas it is greater in elderly individuals due to osteoporosis. In this study, the incidence of healing with shortening of the femoral neck was 46.9%, which was greater than that in young and middle-aged individuals. Correlation analysis revealed that healing with shortening of the femoral neck was positively correlated with age and the Singh index, *i.e*., the older the patient was and the higher the Singh index level was (indicating more severe osteoporosis), the greater was the degree of healing with femoral neck shortening. AVN is an unsolved problem in patients with FNFs and is closely related to local blood supply disorders. However, the incidence of AVN in this study was not greater than that in young and middle-aged individuals. The risk factors for AVN in patients with Pauwels type III FNFs and various individual factors, such as obesity, drinking, and smoking, include increased intraarticular pressure and delayed reduction of fracture displacement [23-27]. In this study, fixation with PCCPs was indicated for elderly adults with displaced FNFs, who were in relatively good physical condition and had no severe cases of osteoporosis (Singh index level IV and above). For all of the healed fractures, the incidences of AVN and delayed healing were 9.4% and 6.3%, respectively, all of which were related to displaced subcapital or transcervical type and Pauwels type III FNFs. The correlation analysis revealed that AVN was positively correlated with Pauwels classification (*p* < 0.05), *i.e*., the higher the Pauwels classification was, the higher the AVN rate. These outcomes support the view in the literature that Pauwels type III FNFs, especially displaced fractures, are risk factors for AVN [26-30].

## 5 LIMITATIONS AND STRENGTHS

The limitations of this study were as follows: (1) it was a retrospective study involving patients from only two hospitals, thereby resulting in a small number of patients for examination; (2) the incidence of AVN may increase with time. Therefore, the results of this investigation need to be further verified in multicenter, prospective studies with long-term follow-up [31]. However, this study investigated the outcomes of PCCP fixation for displaced FNFs in elderly patients; consequently, the present findings can provide a reference for selecting treatment methods for these patients.

## CONCLUSION

The efficacy of PCCPs for the internal fixation of displaced FNFs in elderly individuals without severe osteoporosis is satisfactory, especially for patients who can ambulate early after the operation. The main complications are healing with shortening of the femoral neck and AVN, which are prone to occur in patients with severe osteoporosis and Pauwels type III FNFs, respectively.

## Figures and Tables

**Fig. (1):**
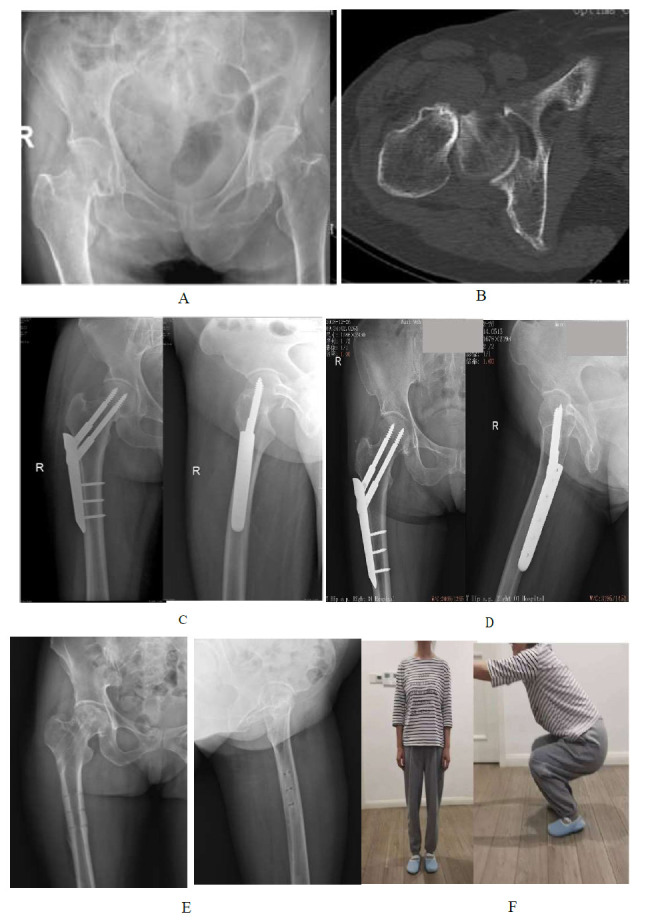
A 67-year-old female patient with a right displaced FNF treated with a PCCP. **A**, **B**: Preoperative radiograph and CT image showing a displaced right FNF with Singh index level V (subcapital type). **C**: Radiographs at week 1 after the operation showing anatomical reduction. **D**: Radiographs at 1 year after the operation showing that the fractures had healed. **E**: Radiographs at 2 years after the operation showing the removal of the internal fixator. **F**: Appearance diagram of the patients at the last follow-up, who recovered with an HHS of 96 points, which was excellent.

**Table 1:** Relationships between treatment efficacy and age, Pauwels classification, Singh index, and Garden alignment index.

Variable	Number (cases)	Delayed Healing	Healing with Femoral Neck Shortening	AVN	Functional Recovery			
					Excellent	Good	Fair	Poor
Age(years)	-	-						
65~69 70~74 >75	15 10 7	2 1 0	2 6 5	1 1 1	6 7 1 1 6 2 1 1 3 3 1 0			
Statistics	-	Z=1.005 *p*=0.605	Z=8.942 *p*=0.011	Z=0.333 *p*=0.847	Z=0.115 *p*=0.908			
Pauwels classification	-	-						
I II III	6 15 11	0 2 0	5 5 3	0 0 3	4 2 0 0 6 7 2 0 3 5 3 0			
Statistics	-	Z=0.556 *p*=0.757	Z=5.681 *p*=0.058	Z=6.320 *p*=0.042	Z=1.105 *p*= 0.269			
Singh index	-	-						
IV V VI	12 14 6	0 2 0	1 6 6	1 1 1	6 4 2 0 7 6 1 0 2 2 2 0			
Statistics	-	Z=2.742 *p*=0.254	Z=13.986 *p*=0.001	Z=0.472 *p*=0.789	Z=0.805 *p*=0.421			
Garden alignment index	-	-						
I II III	25 6 1	2 0 0	13 2 0	2 1 0	11 10 4 0 4 1 1 0 0 1 0 0			
Statistics	-	Z=0.597 *p*=0.742	Z=1.588 *p*=0.452	Z=0.535 *p*=0.755	Z=0.507 *p*=0.612			

## Data Availability

The datasets used and/or analyzed during the current study are available from the corresponding authors [H.L] and [Y.L] upon reasonable request.

## LIST OF ABBREVIATIONS

PCCP: Percutaneous compression plate
FNF: Femoral neck fracture
CS: Compression screw
DHS: Dynamic hip screw
AVN: Avascular necrosis
HHS: Harris hip score

## STANDARDS OF REPORTING

STROBE guidelines were followed.
